# Pulmonary Hypertension in Wild Type Mice and Animals with Genetic Deficit in K_Ca_2.3 and K_Ca_3.1 Channels

**DOI:** 10.1371/journal.pone.0097687

**Published:** 2014-05-23

**Authors:** Christine Wandall-Frostholm, Lykke Moran Skaarup, Veeranjaneyulu Sadda, Gorm Nielsen, Elise Røge Hedegaard, Susie Mogensen, Ralf Köhler, Ulf Simonsen

**Affiliations:** 1 Department of Biomedicine, Aarhus University, Aarhus, Denmark; 2 Institute for Molecular Medicine, Cardiovascular and Renal Research, University of Southern Denmark, Odense, Denmark; 3 Aragon Institute of Health Sciences I+CS and ARAID, Zaragoza, Spain; Indiana University, United States of America

## Abstract

**Objective:**

In vascular biology, endothelial K_Ca_2.3 and K_Ca_3.1 channels contribute to arterial blood pressure regulation by producing membrane hyperpolarization and smooth muscle relaxation. The role of K_Ca_2.3 and K_Ca_3.1 channels in the pulmonary circulation is not fully established. Using mice with genetically encoded deficit of K_Ca_2.3 and K_Ca_3.1 channels, this study investigated the effect of loss of the channels in hypoxia-induced pulmonary hypertension.

**Approach and Result:**

Male wild type and K_Ca_3.1^−/−^/K_Ca_2.3^T/T(+DOX)^ mice were exposed to chronic hypoxia for four weeks to induce pulmonary hypertension. The degree of pulmonary hypertension was evaluated by right ventricular pressure and assessment of right ventricular hypertrophy. Segments of pulmonary arteries were mounted in a wire myograph for functional studies and morphometric studies were performed on lung sections. Chronic hypoxia induced pulmonary hypertension, right ventricular hypertrophy, increased lung weight, and increased hematocrit levels in either genotype. The K_Ca_3.1^−/−^/K_Ca_2.3^T/T(+DOX)^ mice developed structural alterations in the heart with increased right ventricular wall thickness as well as in pulmonary vessels with increased lumen size in partially- and fully-muscularized vessels and decreased wall area, not seen in wild type mice. Exposure to chronic hypoxia up-regulated the gene expression of the K_Ca_2.3 channel by twofold in wild type mice and increased by 2.5-fold the relaxation evoked by the K_Ca_2.3 and K_Ca_3.1 channel activator NS309, whereas the acetylcholine-induced relaxation - sensitive to the combination of K_Ca_2.3 and K_Ca_3.1 channel blockers, apamin and charybdotoxin - was reduced by 2.5-fold in chronic hypoxic mice of either genotype.

**Conclusion:**

Despite the deficits of the K_Ca_2.3 and K_Ca_3.1 channels failed to change hypoxia-induced pulmonary hypertension, the up-regulation of K_Ca_2.3-gene expression and increased NS309-induced relaxation in wild-type mice point to a novel mechanism to counteract pulmonary hypertension and to a potential therapeutic utility of K_Ca_2.3/K_Ca_3.1 activators for the treatment of pulmonary hypertension.

## Introduction

Pulmonary hypertension is a disabling disease with increased blood pressure in the pulmonary circulation, resulting in shortness of breath, dizziness, edema, right ventricular hypertrophy, heart failure, and premature death. Pulmonary hypertension involves endothelial dysfunction, vasoconstriction, and remodeling of the pulmonary vasculature. The current treatment options are unsatisfactory [1].

The endothelium participates in regulating vascular tone by releasing vasoactive autacoids. Endothelium-dependent relaxation involves the release of a variety of diffusible relaxing factors such as nitric oxide (NO) [2], [3], prostacyclin [4], CYP450-generated epoxyeicosanoids acids (11,12-EET and 14,15-EET) [5]–[7], and an electrical mechanism known as endothelium-derived hyperpolarization (EDH) [8]–[10] that produces hyperpolarization of the underlying smooth muscle and closure of voltage-gated calcium channels. The resulting reduction of intracellular calcium leads to relaxation. The small-conductance and intermediate-conductance calcium-activated potassium channels, K_Ca_2.3 and K_Ca_3.1 channel, respectively [11], have been demonstrated to play a significant role in EDH-type relaxation, as a combination of the K_Ca_2.3 and K_Ca_3.1 channel blockers (apamin plus charybdotoxin or apamin plus TRAM-34) inhibits EDH-type relaxation [8], [12]–[14]. Impaired EDH-mediated relaxation involving the K_Ca_2.3 and K_Ca_3.1 channels have been reported to contribute to endothelial dysfunction associated with various human and experimental cardiovascular disease such as hypertension, diabetes, and restenosis [15]–[18]. In line with the roles of the two channels in endothelial function, K_Ca_3.1^−/−^ mice have been reported to have moderately elevated blood pressure [19] and to develop mild left ventricular hypertrophy, as well as a pronounced defect of endothelium-dependent acetylcholine-induced relaxation in both carotid artery and resistance vessels [19], [20]. Likewise, suppression of K_Ca_2.3 gene expression in K_Ca_2.3^T/T(+DOX)^ mice has been shown to elevate blood pressure and increase pressure- and phenylephrine-induced constrictions [21]. Combined K_Ca_3.1- and K_Ca_2.3 channel deficiency in transgenic doxycycline-treated K_Ca_3.1^−/−^/ K_Ca_2.3^T/T(+DOX)^ mice has been reported to increase blood pressure, reduce hyperpolarization of endothelial cells, and to reduce acetylcholine-induced relaxation in carotid arteries and resistance arteries, although the combined deficiency of the two channels has no additive effects on blood pressure [22]. While the roles of the K_Ca_2.3- and the K_Ca_3.1 channels have been at least partially elucidated for systemic blood pressure control [19], [21]–[23], the precise role of the channels in the pulmonary circulation is still unclear.

Thus far, K_Ca_2.3 and K_Ca_3.1 expression have been found in human, porcine, and rat pulmonary artery endothelial cells [24]–[27], as well as in the bronchial epithelium [26], [27]. In pulmonary arteries relaxation sensitive to blockers of the K_Ca_2.3 and K_Ca_3.1 channels has been observed in several studies [26], [28]–[30]. Moreover, the potent K_Ca_2.3- and K_Ca_3.1 channel activator NS309 (6,7-dichloro-1H-indole-2,3-dione 3-oxime) has been shown to induce relaxation being sensitive to K_Ca_2.3- and K_Ca_3.1 blockade in rat and human pulmonary arteries [26], [27], rat mesenteric arteries [31], and in porcine retinal arterioles [32]. In addition, K_Ca_3.1 expression has also been found in proliferating vascular smooth muscle cells [33], [34], and it may also be increased in the smooth muscle of the hypertrophied arteries in pulmonary hypertension.

In this study, we used K_Ca_3.1^−/−^/K_Ca_2.3^T/T(+DOX)^ mice to study the physiological role of K_Ca_2.3 and K_Ca_3.1 channels in the pulmonary arteries from mice with hypoxia-induced pulmonary hypertension. Our hypothesis was that K_Ca_2.3 and K_Ca_3.1 channels participate in maintaining endothelial function in the pulmonary vasculature, whereas deficiency of K_Ca_2.3- and K_Ca_3.1 channels aggravates endothelial dysfunction, and thereby contributes to pulmonary hypertension and the associated structural pathologies. Moreover, we tested whether activation of the channels by NS309 improves endothelium-dependent vasodilatation in pulmonary arteries of mice with hypoxia-induced pulmonary hypertension.

## Materials and Methods

### Ethics statement

This study followed the recommendations in the Guide for the Care and Use of Laboratory Animals of the National Institutes of Health and the ARRIVE Guidelines. The animal studies were approved by the Animal Ethical Committee according to Danish legislation (permit no. 2011/561-2011).

### Animal model

The mice were housed in standard cages and had free access to water and food. K_Ca_3.1^−/−^/K_Ca_2.3^T/T^ mice were generated by interbreeding of K_Ca_3.1^−/−^ [19] and K_Ca_2.3^T/T^ [35] mice as previously described [22]. 32 Male C57Bl/6 mice and K_Ca_3.1^−/−^/ K_Ca_2.3^T/T^ mice, with an age between 15–25 weeks, were given doxycycline (DOX) (Piggidox, 2 mg/kg) in the drinking water, six days before the start of the experiment and throughout the experiment for the suppression of the K_Ca_2.3 channel in the K_Ca_3.1^−/−^/ K_Ca_2.3^T/T^ mice. Each genotype was divided into normoxic and hypoxic groups. The hypoxic groups were placed in hypobaric chambers and exposed to hypoxia for four weeks to induce pulmonary hypertension. The hypobaric chambers were connected to a vacuum pump and depressurized to 560 mbar corresponding to an atmospheric oxygen tension of 10%, as previously described for rats [36].

### Hemodynamic measurements

The animal was weighed and anaesthetized by an intraperitoneal injection with a combination of fentanyl (3.313 mg/kg), fluanisone (104.8 mg/kg), and midazolam (52.44 mg/kg), and if necessary the anesthesia was maintained every 30 min. The neck area was shaved and the animal was placed on a heating pad. A 1 cm incision was made on the right side of the neck. By blunt dissection, the right external jugular vein was located and isolated. A hole was cut in the vein and through this, a catheter (SPC-1000, Millar Instruments Inc, USA) was inserted and secured by a suture. The catheter was led into the right ventricle and the position was confirmed by observing the characteristic ventricular waveform. The pressure profile was recorded over a three minute period using a Quad Bridge amplifier connected to a PowerLab device (ADInstruments, Oxfordshire, England) and recorded with Chart 5.5 (ADInstruments). The animal was euthanized after blood pressure measurement by cervical dislocation.

### Assessment of right ventricular hypertrophy

Immediately after the mice were sacrificed, heart, lungs, and liver were removed and kept in cold (5° C) physiological saline solution (PSS). A blood sample was collected in a microcapillary tube and centrifuged (International equipment company, micro-capillary centrifuge model M B) for three hours as previously described [36].

The hematocrit value was calculated as the length of the erythrocyte layer divided by the length of the entire blood sample. Wet weight of liver and left lung were determined and expressed as percentage of body weight. The heart was isolated and both atria were removed. The free wall of the right ventricle (RV) was separated from left ventricle and septum (LV+S). Each part was weighed and the ratios of right ventricle to left ventricle plus septum (RV/(LV+S)) were calculated and used to assess right ventricular hypertrophy. The thickness of the right ventricular free wall, as well as the septum was measured with a Zeiss Eyepiece calibrating reticule at three different points, at the middle of the wall, and each half then bisected in a cranial and caudal direction, respectively.

### QPCR

Samples of lung tissue were dissected and placed in RNAlater filled Eppendorff tubes at 4°C for 24 hours and then stored at −20°C. RNA was extracted using Trizol reagent (Invitrogen, Naerum, Denmark), and genomic DNA was eliminated using DNAse I (Qiagen Germantown, MD). The concentration of RNA was determined densitometrically at 260 nm. Reverse transcription of RNA was performed using iScript cDNA synthesis kit (Bio-Rad, Copenhagen, Denmark). A standard PCR protocol was used with initial denaturing at 95°C for 3 min and subsequently 40 cycles of denaturing at 95°C for 25 sec, annealing at 57°C for 20 sec and extending at 72°C for 40 sec. A final step of extension was done at 72°C for 3 min. The PCR-products were analyzed by gel electrophoresis using 1.5% agarose in Tris-borate-EDTA buffer and staining with GelRed™ (Biotium, Hayward, CA). Semiquantitative RT-PCR was done using iQ™ CYBR® Green Supermix (Bio-Rad) and a Stratagene MX3000p cycler (Stratagene, La Jolla, CA) with an initial denaturing at 95°C for 10 min followed by 40 cycles of denaturing at 95°C for 20 sec, annealing at 60°C for 20 sec and an extension step at 72°C for 20 sec. Thereafter, a three-step series of 95°C for 60 sec, 55°C for 30 sec, and a gradual increase to 95°C was run to determine melting points of qPCR products. Signals were normalized to expression of the house keeping gene clathrin (CLCT) and are given as % CLCT. The identity of all PCR products was verified by sequencing. Specific RT-PCR primers were designed to span intronic sequences. The expected product lengths were 226 bp for K_Ca_2.1, 250 bp for K_Ca_2.2, 70 bp for K_Ca_2.3, 217 bp for K_Ca_3.1, 349 for K_Ca_1.1, 416 bp for endothelial nitric oxide synthase (eNOS), 152 bp for smooth muscle actin (SMA), 176 bp for collagen-1, and 150 bp for transforming growth factor β (TGFβ). Primer sequences were as follows (5′ → 3′): K_Ca_2.1 FP, TCAAAAATGCTGCTGCAAAC; K_Ca_2.1 RP, TCATATGCGATGCTCTGGTGC; K_Ca_2.2 FP, GGTCATTGAGACCGAGCTGT; K_Ca_2.2 RP, ATTCCCAGGTATGGGATGGA; K_Ca_2.3 FP, CCATGCCAAAGTCAGGAAAC; K_Ca_2.3 RP, CATCTTGACACCCCGAAGTT; K_Ca_3.1 FP, CTGAGAGGCAGGCTGTCAATG; K_Ca_3.1 RP, ACGTGTTTCTCCGCCTTGTT; K_Ca_1.1 FP, ACACTTGGACGCCTCTTCAT; K_Ca_1.1 RP, CTCTGGCAAGATCATGTGGA; eNOS FP, GGTGTCCCTAGAGCACGA; eNOS RP, CTCGGAAAGCCTCCTCCT; SMA FP, AATGGCTCTGGGCTCTGTAA; SMA RP, CTCTTGCTCTGGGCTTCATC; Collagen-1 FP, GAACCCCAAGGAAAAGAAGC; Collagen-1 RP, GCTACGCTGTTCTTGCAGTG; TGFβ FP, ACCGGAGAGCCCTGGATAC; TGFβ RP, AGGGTCCCAGACAGAAGTTG; CLCT FP, AAGGAGGCGAAACTC ACAGA; CLCT RP, GAGCAGTCA ACATCCAGCAA.

In previous studies we have performed immunoblotting of lungs from rat and man [26], [27], but unfortunately we were unable to validate the gene expression results in this study with protein quantification in mouse lung using western blotting, as we experienced a series of difficulties (e.g. lack of specificity and multiple bands) with the mouse antibodies for the K_Ca_2.3 and K_Ca_3.1 channels, despite trying several types (K_Ca_2.3: SC-28621 (Santa Cruz Biotechnology, Santa Cruz, CA); APC-025 (Alamone Labs, Jerusalem, Israel); H00003782-A01 (Abnova, Taiwan). K_Ca_3.1: P4997, AV35098 (Sigma-Aldrich, St. Louis, MO); SC-32949 (Santa Cruz Biotechnology); CA1788 (Cell Applications, San Diego, CA). All mice were genotyped and QPCR performed as stated above.

### Genotyping

All animals were tested for the presence of the K_Ca_3.1^−/−^ and K_Ca_2.3^T/T^ alleles by polymerase chain reaction (PCR) on DNA extracts from tail tips. The reaction was carried out in a thermal cycler (ABI system, Applied Biosystems, Carlsbad, CA) using the following protocol, an initial denaturation step at 94°C for 3 min, followed by 10 cycles consisting of 35 sec at 94°C, 35 sec at 58°C and 50 sec at 72°C, proceeded by 25 cycles of the same three steps at the same temperature but with additional 5 sec prolongation at the extension step per cycle, and a final extension step (10 min at 72°C). The reaction product was analysed on a 1% agarose gel. The primer sequence were as follows K_Ca_2.3^T/T^: Forward primer (FP) 5′-ATGGACACTTCTGGGCACTT-3′, Reverse Primer (RP) 5′-AGAGTGCAACAGACCAGGAT; K_Ca_3.1^+/+^/K_Ca_3.1^-/-^ : FP 5′-CTTTGGATCCAGATGTTTCTTGGTG- 3′, K_Ca_3.1^+/+^ RP 5′-GCCACAGTGTGTCTGTGAGG-3′; K_Ca_3.1^-/-^ RP 5′- CGTGCAATCCATCTTGTTCA-3′.

### Lung perfusion and immunohistochemistry

Catheters with heparin (0.1 ml 100 IU, LEO Pharma, Copenhagen, Denmark) were inserted into the truncus pulmonalis as well as in the trachea and secured by sutures. The left lung lobe was first perfused with a perfusion mixture (calcium free PSS, 2% albumin, 10% heparin (100 IU), and papaverin (10^−4^ M; Sigma-Aldrich)) and then with 4% formaldehyde. The perfusion pressure was maintained at 12–14 mmHg using fluid columns. The left lung lobe was weighed and fixed in 4% formaldehyde for two days and was kept afterwards in ethanol (70%) until embedding in paraffin. The paraffin-embedded left lung lobe was cut in transverse sections of 5 µm. The sections were de-paraffinized and rehydrated by washing 2×5 min each in: xylene, a decreasing ethanol series (99, 96, 76%), and distilled water. Antigen retrieval was performed using a Tris/EDTA pH 9.0 buffer and heating in a microwave for 2×5 min/600 W. Endogenous peroxidase activity was blocked by incubation with 3% H_2_O_2_ for 20 min, followed by washing for 2×5 min in PBS. To avoid non-specific binding of primary antibodies, sections were incubated with 10% calf serum for 10 min. Thereafter, sections were incubated at 4°C overnight with anti-Von Willebrand factor (1∶500, Dako, Denmark). After washing 2×5 min in PBS, sections were incubated in HRP-conjugated secondary antibody (goat-anti rabbit, 1∶3000, Zymed laboratories, USA) for one hour at room temperature and washed again 2×5 min in PBS. DAB chromogen (Sigma, USA) was added and sections were incubated for 5 min. Sections were washed again 4×5 min in PBS and incubated with 10% calf serum for 10 min before incubating with primary anti-α-smooth muscle actin (1∶250, Abcam, UK) for 75 min at room temperature. After wash 2×5 min in PBS, sections were incubated in AP-conjugated secondary antibody (donkey-anti goat, 1∶500, Abcam, UK) one hour at room temperature and washed again 2×5 min in PBS. Stay red chromogen (Sigma-Aldrich) was added and incubated for 5–10 min. After 5 min wash in distilled water, sections were incubated with Mayer's hematoxylin for 20 sec, washed again in water and mounted using **Flourmount mounting media** (Sigma-Aldrich).

### Morphometric measurements

At 400× magnification small pulmonary vessels of each animal ranging from 10 to 80 µm in internal diameter were counted in a blinded manner. Non-muscular arterioles were detected by the endothelial anti-Von Willebrand staining. To assess the degree of muscularization, the amount of α-SMA-positive vessel wall area was determined. Arteries that contained α-SMA-positive vessel area between 4 and 69% were defined as partially-muscularized. Arteries that contained 70% and above were defined as fully-muscularized. Wall area was expressed as percentage of the vessel using the following formulae: % wall area  =  ([area_outer_ – area_inner_) / area_outer_) ×100. The area was calculated using the two perpendicular diameters for both vessel (diameter_outer_) and lumen (diameter_inner_) and applying the area formulae for ellipses (area_ellipse_  =  π × the two perpendicular radiuses) to account for deviations from the perfect circle form. To avoid oblique sections, vessels with an area difference of more than 20% between the areal calculated as ellipse and circle (area_circle_  =  π × radius^2^) were excluded.

### Functional studies

Segments of 2^nd^ and 3^rd^ order pulmonary artery (approximately 2 mm in length) with an internal diameter of 564±28 µm (n = 55) in wild type mice and of 534±20 µm (n = 58) in K_Ca_3.1^−/−^/K_Ca_2.3^T/T(+DOX)^ mice (n.s. vs. wt) were dissected from the right lung lobes and mounted on two 40 µm wires in microvascular myographs (Danish Myotechnology, Aarhus, Denmark) for isometric tension recordings. The myograph chamber filled with PSS was heated to 37° C and equilibrated with 5% CO_2_ in artificial air (20.9% O_2_, 74% N_2_). The artery segments were stepwise stretched to 2.4 kPa, corresponding to a transmural pressure of 18 mmHg. The segments were allowed to equilibrate for about 10 min. The viability of the segments was confirmed by their ability to contract to first potassium-rich PSS (KPSS, 60 mM) and subsequently to phenylephrine (PE, 1 µM). All arteries were incubated with the cyclooxygenase inhibitor indomethacin (3 µM) for 30 min before concentration-response curves were constructed. One artery segment from each mouse was incubated with the eNOS inhibitor *N* ^G^-nitro-L-arginine (L-NNA, 10^−4^ M) for 30 min, another segment was incubated with a combination of apamin (5*10^−7^ M) and charybdotoxin (ChTx, 10^−7^ M) for 15 min, and a third artery segment was kept as a control with no additional inhibitors. The arteries were contracted with PE (10^−7^ M) and upon stable contraction, acetylcholine (3*10^−7^ M) was added. After a washout and incubation with the respective blockers, the arteries were contracted with PE (10^−7^ M) and when stable contraction was obtained, NS309 concentration-response curves (10^−8^–3*10^−5^ M) were performed. After a washout and incubation with the blockers, the arteries were contracted to PE (10^−7^ M) and when the contraction was stable, sodium nitroprusside (SNP) concentration-response curves (10^−10^–10^−5^ M) were performed. The response of the artery segments was measured as the change in force. The data on relaxation are given as percentage (% relaxation) of the contraction induced by PE.

### Drugs and solutions

Doxycycline (Piggidox Vet.) was obtained from Boehringer Ingelheim Danmark A/S). Hypnorm (fentanyl citrate 0.315 mg/ml, fluanisone 10 mg/ml) was purchased from VetaPharma Ltd (Leeds, UK) and dormicum (midazolam, 5 mg/ml) from Hoffmann-La Roche (Basel, Switzerland).

#### For myograph experiments

The PSS was of the following composition (mM): CaCl_2_ 1.6, NaCl 119, KCl 4.7, glucose 5.5, MgSO_4_ H_2_O 1.17, NaHCO_3_ 25, KH_2_PO_4_ 1.18, and EDTA 0.026. The calcium-free PSS solution was of the same composition without CaCl_2_ 1.6, and potassium-rich PSS (KPSS) solution was of the same composition with NaCl exchanged with KCl to give a final concentration 60 mM. Acetylcholine, indomethacin,PE; L-NNA, and SNP were purchased from Sigma-Aldrich (St. Louis, MO). Apamin and charybdotoxin were purchased from Latoxan (Valence, France). NS309 was kindly donated from Neurosearch (Ballerup, Denmark). For preparation of stock solutions, all drugs were dissolved in distilled water, with the exception of indomethacin and NS309 that were dissolved in DMSO and further diluted in PSS to achieve the final concentration. Apamin and ChTx were prepared in albumin-coated tubes. All solutions were kept at −20° C until use.

### Statistics

The computer program GraphPad Prism (San Diego, CA, USA) was used for statistical analysis. Data were expressed as means ±SEM and differences between groups were analyzed by using two-way ANOVA followed by Bonferroni post hoc test. A probability value of P<0.05 was considered significant. The data from a vessel was excluded from the dataset due to technical reasons.

## Results

### Basic characteristics of normoxic and hypoxic strains

Body weight, heart rate, hematocrit, heart weight-, wet lung weight- and liver weight normalized to body weight are presented in Table 1. Age matched K_Ca_3.1^−/−^/K_Ca_2.3^T/T(+ DOX)^ mice were smaller than wild type mice. The body weight of chronic hypoxic mice of either genotype was smaller compared to normoxic mice. Exposure to chronic hypoxia increased hematocrit levels, wet lung weight and liver weight. The chronic hypoxic K_Ca_3.1^−/−^/K_Ca_2.3^T/T(+DOX)^ mice however had a significantly smaller increase in liver weight than the chronic hypoxic wild type mice. The heart weight remained unaltered in the normoxic/chronic hypoxic wild type- and K_Ca_3.1^−/−^/K_Ca_2.3^T/T(+DOX)^ mice.

**Table 1:** Characteristics of the animals.

		Wild type	K_Ca_3.1^−/−^/K_Ca_2.3^T/T(+DOX)^
Initial	Normoxia	33±1.68	25±0.61*
body weight (g)	Hypoxia	32±1.35	23±0.65*
End	Normoxia	34±1.11	25±0.77*
body weight (g)	Hypoxia	30±0.76#	21±0.56*#
Heart rate	Normoxia	466±33	393±30
(BPM)	Hypoxia	413±48	402±27
Hematocrit	Normoxia	39±3.01	38±3,20
(%)	Hypoxia	54±2.38#	55±3.64#
Heart weight	Normoxia	0.5±0.026	0.5±0.017
(% of BW)	Hypoxia	0.5±0.015	0.5±0.020
RV	Normoxia	0.10±0.003	0.11±0.005
(% of BW)	Hypoxia	0.14±0.006#	0.14±0.006#
LV+S	Normoxia	0.40±0.027	0.39±0.011
(% of BW)	Hypoxia	0.36±0.012	0.36±0.018
Lung weight	Normoxia	0.3±0.047	0.4±0.062
(% of BW)	Hypoxia	0.5±0.059#	0.5±0.063#
Liver weight	Normoxia	4.9±0.69	4.9±0.14
(% of BW)	Hypoxia	6.0±0.21#	5.5±0.16#

Selected morphological and functional characteristics and hematocrit of the normoxic and hypoxic strains. Values are means ±SEM, n = 7–8. BPM =  beats per minute. BW =  body weight. *P<0.05 vs. wild type;

# P<0.05 vs. normoxia; 2-way ANOVA (n = 7–8 per group).

### Hemodynamics

**Figure 1:**
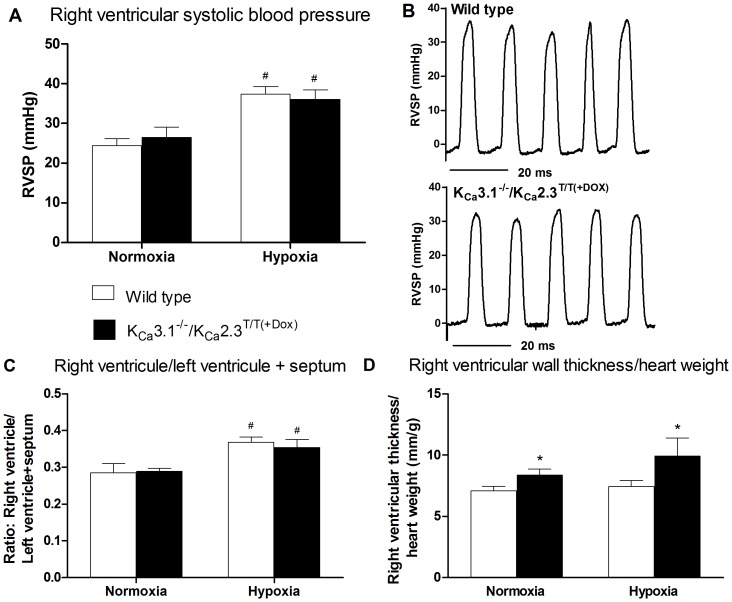
Right ventricular systolic blood pressure and hypertrophy. A) The effect of chronic hypoxia on right ventricular systolic blood pressure (RVSBP) in wild type and K_Ca_3.1^−/−^/K_Ca_2.3^T/T(+Dox)^ mice. Values are means ±SEM, normoxic wild type (n = 8) and K_Ca_3.1^−/−^/K_Ca_2.3^T/T(+Dox)^ mice (n = 7). B) Representative trace of right ventricular pressure measurements in normoxic wild type mice (top) and normoxic K_Ca_3.1^−/−^/K_Ca_2.3^T/T(+Dox)^ mice (bottom). C) Hypoxia induced right ventricular hypertrophy as indicated by alterations of the weight ratio of right ventricle/left ventricle + septum, in wild type and K_Ca_3.1^−/−^/K_Ca_2.3^T/T(+Dox)^ mice, n = 8. D) The effect of hypoxia on right ventricular wall thickness/heart weight (HW) in wild type and K_Ca_3.1^−/−^/K_Ca_2.3^T/T(+Dox)^ mice. Values are mean ±SEM, n = 8. Data were analyzed by 2−way ANOVA and differences were considered significant when *P<0.05 vs. wild type, ^#^ P<0.05 vs. normoxia.

Exposure to chronic hypoxia increased the right ventricular systolic blood pressure in normoxic mice of either genotype by 10 to 12 mm Hg (from 24.3±1.8 mmHg to 37.4±1.9 mmHg in wild type mice and from 26.4±2.6 mmHg to 36.0±2.4 mmHg in K_Ca_3.1^−/−^/K_Ca_2.3^T/T(+DOX)^ mice) and there were no differences between genotypes (Fig. 1A for means and Fig. 1B for tracings). Heart rate was not affected by chronic hypoxia in either genotype and there were no differences between genotypes (Table 1).

### Right ventricular hypertrophy

Right ventricular hypertrophy was assessed by determining the weight ratio of the right ventricle over the left ventricle plus septum (RV/LV+S). The chronic hypoxic mice developed right ventricular hypertrophy compared to normoxic mice (Fig. 1C). There was no difference in the degree of right ventricular hypertrophy between wild type- and K_Ca_3.1^−/−^/K_Ca_2.3^T/T(+DOX)^ mice in hypoxia. However, hypoxic K_Ca_3.1^−/−^/K_Ca_2.3^T/T(+DOX)^ mice had an increased right ventricular wall thickness compared to wild type mice (Fig. 1D).

### mRNA expression studies

**Figure 2:**
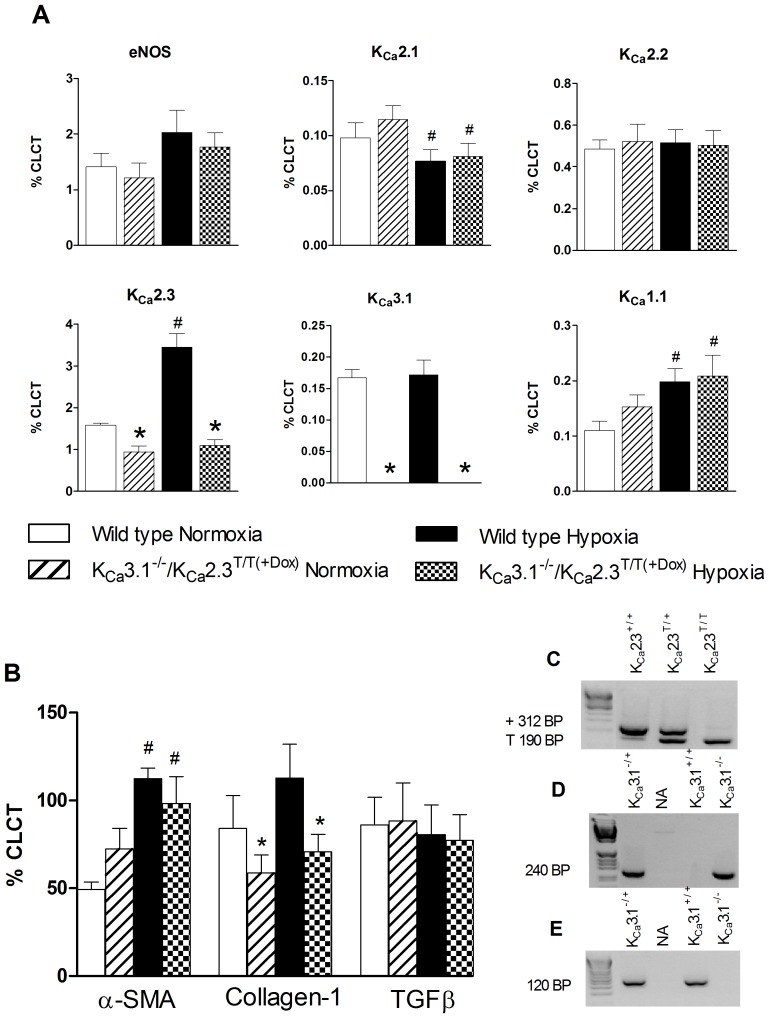
Lung mRNA expression levels of A) eNOS, K_Ca_2.1, K_Ca_2.2, K_Ca_2.3, K_Ca_3.1, and K_Ca_1.1; B) α-Smooth muscle actin (α-SMA), collagen-1, and TGFβ. Data are given as means ±SEM, n = 7–8. Data were analyzed by two-way ANOVA and differences were considered significant when *P<0.05 from wild type, ^#^ P<0.05 from normoxia. Statistical interaction (£) was observed in K_Ca_2.3 expression (A). C–E) Genotyping: C:Gel electrophoresis shows that polymerase chain reacton (PCR) detected the K_Ca_2.3-wild type allele (wild type (+)) and the tTA allele (T) in K_Ca_2.3^T/+^, and K_Ca_2.3^T/T^. D and E: PCR detected the targeted allele in K_Ca_3.1^−/+^ and in K_Ca_3.1^−/−^ as well as the wild type allele in K_Ca_3.1^+/−^ and K_Ca_3.1^+/+^. A DNA ladder was used to determine products sizes.

As shown in Fig. 2A, quantitative RT-PCR confirmed the loss of K_Ca_3.1-exon 4 transcripts and reduced K_Ca_2.3 mRNA expression in the K_Ca_3.1^−/−^/K_Ca_2.3^T/T(+DOX)^ mice as expected. In wild type mice, exposure to chronic hypoxia up-regulated K_Ca_2.3 and K_Ca_1.1 mRNA expression, whereas K_Ca_2.1 channel expression was down-regulated. With the exception of K_Ca_2.3, mRNA expression of K_Ca_1.1 was also increased in the K_Ca_3.1^−/−^/ K_Ca_2.3^T/T(+DOX)^ and K_Ca_2.1 expression was down-regulated in a similar fashion. Neither genotype nor hypoxia affected mRNA expression of K_Ca_2.2 or eNOS.

As shown in Fig. 2B, expression of α-smooth muscle actin (α-SMA) was increased in hypoxic wild type- and K_Ca_3.1^−/−^/K_Ca_2.3^T/T(+DOX)^ mice if compared to the respective normoxic controls. Expression levels of collagen-1 were not altered by hypoxia but were down-regulated in K_Ca_3.1^−/−^/K_Ca_2.3^T/T(+DOX)^ mice compared to wild type mice. TGFβ expression was not altered by hypoxia or genotype.

### Morphological alterations

**Figure 3:**
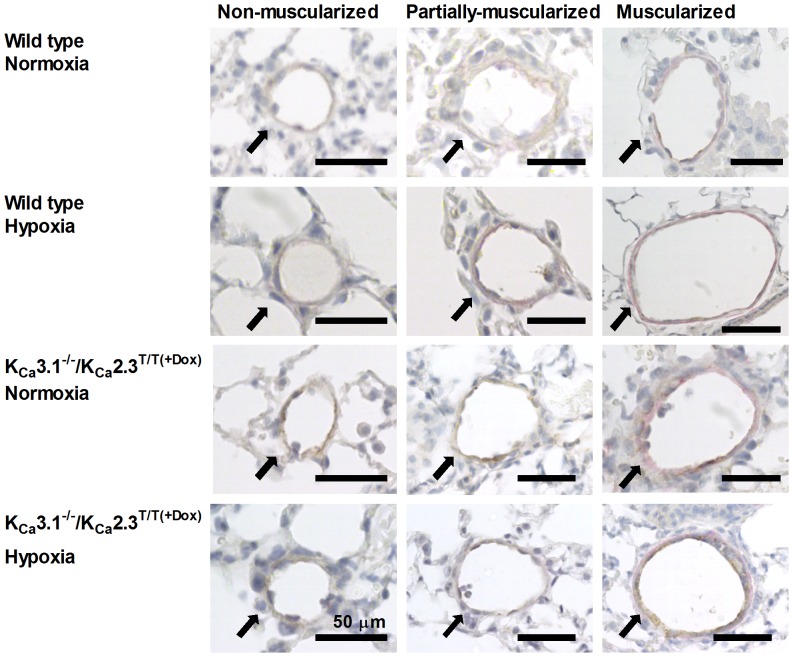
Images of mouse lung showing pulmonary vessels stained for von-Willebrand factor and α-smooth muscle actin from K_Ca_3.1^−/−^/ K_Ca_2.3^T/T(+Dox)^ - and wild type mice under normoxic and hypoxic conditions, identifying non-muscularized vessels, partially-muscularized vessels, and fully-muscularized vessels.

**Figure 4:**
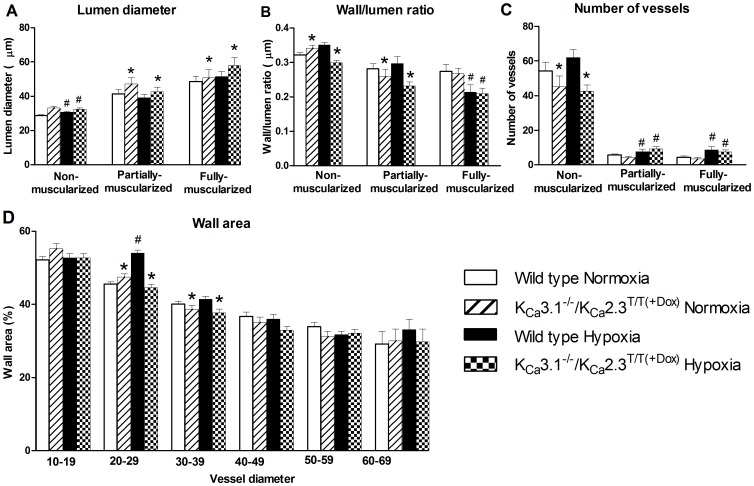
Morphometric measurements. A: Lumen diameter (µm) in vessels divided in groups of non-muscularized-, partially- and fully-muscularized vessels. B: Wall/lumen ratio divided in groups of non-muscularized-, partially- and fully-muscularized vessels. C: Number of non-muscularized-, partially- and muscularized vessels in each experimental group. D: Wall area of the vessels divided in groups of vessel diameter. * P<0.05 vs. wild type and ^#^ P<0.05 vs. normoxia. Statistical interaction was observed in Figure (B) for non-muscularized vessels.

The pulmonary vessels were stained for Von Willebrandt factor and α-smooth muscle actin and non-muscularized, partially-muscularized, and muscularized vessels were counted and measured (Fig. 3). Chronic hypoxic mice of either genotype had significantly decreased lumen diameters of non-muscularized vessels when compared to normoxic mice of either genotype (Fig. 4A). In contrast, chronic hypoxia did not alter the lumen diameters of partially-muscularized- or fully-muscularized vessels of either genotype. However, the partially-muscularized as well as the fully-muscularized vessels in K_Ca_3.1^−/−^/ K_Ca_2.3^T/T(+DOX)^ mice had larger lumen diameter compared to wild type mice.

As shown in Fig. 4B, the wall/lumen ratio for non-muscularized vessels in K_Ca_3.1^−/−^/K_Ca_2.3^T/T(+DOX)^ mice was significantly different from the wild type mice. There was a tendency towards an increased wall/lumen ratio in chronic hypoxic wild type mice if compared to normoxic wild type mice, whereas the ratio was lower in chronic hypoxic K_Ca_3.1^−/−^/K_Ca_2.3^T/T(+DOX)^ mice if compared to normoxic K_Ca_3.1^−/−^/K_Ca_2.3^T/T(+DOX)^ mice. The wall/lumen ratio was decreased in partially-muscularized vessels in K_Ca_3.1^−/−^/K_Ca_2.3^T/T(+DOX)^ mice when compared to wild type mice. Exposure to chronic hypoxia reduced the wall/lumen ratio in muscularized vessels of either genotype.

The numbers of partially and fully muscularized vessels were increased in both wild type and K_Ca_3.1^−/−^/K_Ca_2.3^T/T(+DOX)^ mice (Fig. 4C).

For the analysis of wall area, the data were grouped according to vessel lumen categories (lumen diameters (µm): 10–19; 20–29; 60–69; Fig. 4C) as previously described [37]. In the vessels with a lumen from 20–29 µm the wall area was larger in hypoxic than in normoxic wild type mice. In contrast, the wall area of the vessels from K_Ca_3.1^−/−^/K_Ca_2.3^T/T(+DOX)^ mice was unaltered in response to chronic hypoxia. The wall area was less in vessels with lumen diameters of 20–39 µm in lung sections from K_Ca_3.1^−/−^/K_Ca_2.3^T/T(+DOX)^ mice (Fig. 4D).

### Functional studies

**Figure 5:**
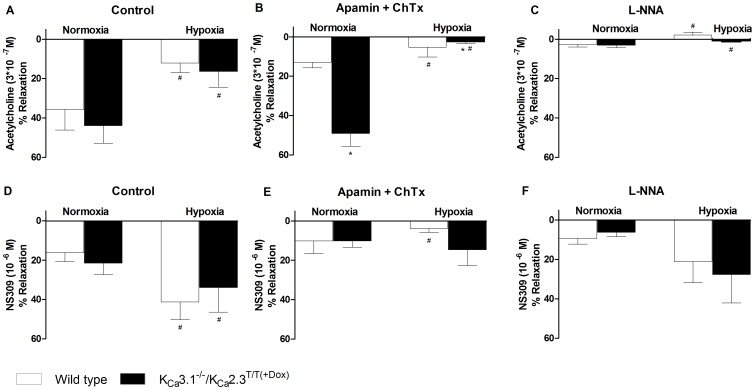
Functional studies of acetylcholine-induced (3*10^−7^ M) relaxation (A–C) and NS309-induced (10^−6^ M) relaxation (D–F) in wild type and K_Ca_3.1^−/−^/K_Ca_2.3^T/T(+Dox)^ mice. A) Acetylcholine-induced relaxation. B) Acetylcholine-evoked relaxation in the presence of apamin (5*10^−7^ M) and ChTx (10^−7^ M). C) Acetylcholine-evoked relaxation in the presence of L-NNA (10^-4^ M). D) NS309-induced relaxation. E) NS309-induced relaxation in the presence of apamin (5*10^−7^ M) and ChTx (10^−7^ M). F) NS309-evoked relaxation in the presence of L-NNA (10^−4^ M). Data were analyzed by 2-way ANOVA and * P<0.05 vs. wild type and ^#^ P<0.05 vs. normoxia; n = 6-7 per group.

Precontracted pulmonary arteries from chronic hypoxic wild type mice exhibited significantly impaired acetylcholine-induced (3*10^−7^ M) relaxation if compared to normoxic wild type mice (Fig. 5A). Chronic hypoxia also reduced acetylcholine-induced relaxation in arteries from K_Ca_3.1^−/−^/K_Ca_2.3^T/T(+DOX)^ mice and responses under either conditions were similar to those in the wild type mice (Fig. 5A). The K_Ca_2.3- and K_Ca_3.1 channel inhibitors, apamin and charybdotoxin reduced the acetylcholine-induced relaxation in both normoxic and chronic hypoxic wild type mice (Fig. 5B). In contrast to wild type mice, apamin and charybdotoxin did not reduce the acetylcholine-induced relaxation in normoxic K_Ca_3.1^−/−^/ K_Ca_2.3^T/T(+DOX)^ mice (Fig. 5B). However, acetylcholine-induced relaxation became sensitive to apamin and charybdotoxin in the hypoxic K_Ca_3.1^−/−^/ K_Ca_2.3^T/T(+DOX)^ mice (Fig. 5B, columns on the right). This might be explained by the up-regulation of mRNA expression of K_Ca_1.1 as described above. Inhibition of NO synthesis by L-NNA almost abolished acetylcholine-evoked relaxation in both normoxic and chronic hypoxic wild type mice as well as in K_Ca_3.1^−/−^/ K_Ca_2.3^T/T(+DOX)^ mice (Fig. 5C), still suggesting that NO is the major endothelium-derived vasodilator in pulmonary arteries of both strains.

NS309 at 10^−6^ M induced similar relaxations of precontracted pulmonary arteries, in both normoxic and hypoxic wild type mice and K_Ca_3.1^−/−^/K_Ca_2.3^T/T(+DOX)^ mice. However, NS309-evoked relaxations were significantly increased in the chronic hypoxic mice of either genotype (Fig. 5D). Incubation with apamin and charybdotoxin did not significantly reduced NS309-induced relaxations of pulmonary arteries from normoxic wild type and K_Ca_3.1^−/−^/K_Ca_2.3^T/T(+DOX)^ mice (Fig. 5E). However, the larger NS309-induced relaxation in hypoxic wild type mice was significantly reduced by apamin and charybdotoxin (Fig. 5E). In contrast, apamin and charybdotoxin failed to reduce the larger NS309-induced relaxations of pulmonary arteries from K_Ca_3.1^−/−^/K_Ca_2.3^T/T(+DOX)^ mice. This suggested that the larger NS309-induced relaxation observed in pulmonary arteries from hypoxic wild mice was mediated by K_Ca_3.1 and K_Ca_2.3 channels. L-NNA reduced NS309-evoked relaxations by trend in both wild type and K_Ca_3.1^−/−^/K_Ca_2.3^T/T(+DOX)^ mice and abolished the difference between normoxic and hypoxic mice (Fig. 5F).

Exposure to chronic hypoxia did not alter the concentration-response curve for SNP (Figure S1). These findings suggest that the smooth muscle response to NO was unaltered.

## Discussion

The main findings of the present study are that the deficits of the K_Ca_2.3 and K_Ca_3.1 channels failed to change hypoxia-induced pulmonary hypertension, and that chronic hypoxia up-regulated K_Ca_2.3-gene expression (and also of K_Ca_1.1) and increased NS309-induced relaxation in wild type mice. The latter finding points to a novel mechanism to counteract pulmonary hypertension and implicates a potential therapeutic usage of K_Ca_2.3/K_Ca_3.1 activators for the treatment of pulmonary hypertension. Moreover, genetic deficit of K_Ca_3.1 channels and partial suppression of K_Ca_2.3 channels caused a significant increase of right ventricular wall thickness and alteration in the pulmonary vessels with reduced wall area of pulmonary vessels and increased lumen diameter in partially-muscularized and fully-muscularized vessels.

### The effects of hypoxia-induced pulmonary hypertension in wild type mice

The hypoxic model of pulmonary hypertension mimics the changes in pulmonary circulation seen in high altitude. Mice exposed to chronic hypoxia develop pulmonary vasoconstriction, pulmonary vascular remodeling, and right ventricular hypertrophy [38].

In this study, the right ventricular systolic blood pressure was significantly increased in the chronic hypoxic mice reflecting pulmonary hypertension. Right ventricular hypertrophy measured as the right ventricle over left ventricle and septum weight ratio, was significantly increased in chronic hypoxic mice, indicating development of pulmonary hypertension in the chronic hypoxic mice.

Our qRT-PCR studies revealed that exposure to chronic hypoxia increased the mRNA expression of K_Ca_2.3 channels by twofold in the lung of wild type mice. Although it appears a limitation of the present study that the increased K_Ca_2.3 expression is found in the lung, it support our previous studies showing up-regulation of K_Ca_2.3 protein expression in pulmonary arteries without alteration in expression in bronchioles from chronic hypoxic rats [39]. Regarding mRNA expression of K_Ca_3.1 channels in animal models of pulmonary hypertension, previous results have been inhomogeneous: K_Ca_3.1 mRNA expression have been found to be up-regulated in rats with monocrotaline-induced pulmonary hypertension [40] or unaltered in chronic hypoxic rats [39]. In the present study on mice, chronic hypoxia did not change the K_Ca_3.1 channel mRNA expression. These discrepancies might be explained by a higher degree of lung fibrosis in monocrotaline-induced pulmonary hypertension than in hypoxia-induced pulmonary hypertension. Development of fibrosis was shown to be accompanied by a higher expression of K_Ca_3.1 [22], [41].

Regarding the NO system, a decrease in production or activity of NO was previously found in chronic hypoxic animals. Several studies demonstrated an increase in eNOS mRNA but a decrease in NO activity [42]–[46], while other studies found that the decreased plasma- and lung perfusate NO^-^_x_ in piglets was related to a decrease in eNOS mRNA rather than decreased activity [47]. In this study, the mRNA expression of eNOS was not significantly changed in the hypoxic mice albeit NO-mediated relaxation of pulmonary arteries in chronically hypoxic mice was reduced as detailed below. This indicates endothelial dysfunction at the level of NO activity and in the absence of compensatory up-regulation of eNOS-mRNA expression.

Hypoxia-induced pulmonary hypertension is associated with vascular remodeling in pulmonary arteries, including the appearance of smooth muscle-like cells in previously non-muscularized vessels, thickening of the media and increased accumulation of smooth muscle cells as well as increased deposition of extracellular matrix proteins, predominantly collagen and elastin [38]. Here we found increased mRNA levels of α-SMA but not of collagen-1 or TGFβ indicating vascular remodeling and media thickening as outlined above but no substantial pulmonary fibrosis in this murine model.

In agreement with previous studies, exposure to chronic hypoxia and the subsequent development of pulmonary hypertension and of endothelial dysfunction were associated with impaired acetylcholine-induced relaxation in wild type mice [48], [49]. The acetylcholine-induced relaxations predominantly involved the NO-pathway as indicated by a full blockade of the response by a blocker of NO-synthesis in hypoxic and normoxic wild type mice. However, also K_Ca_2.3 and K_Ca_3.1 channels contributed to the response in normoxic and hypoxic mice since blockers of the channels clearly reduced the response under either condition.

Regarding the effects of pharmacological activation of the K_Ca_2.3 and K_Ca_3.1 channels in pulmonary arteries, a previous study showed that exposure to chronic hypoxia reduced the relaxations in pulmonary arteries induced by the K_Ca_3.1/K_Ca_2.3 channel activator NS4591[39]. In contrast, in our study, chronic hypoxia increased the relaxation to the activator NS309 by 3-fold. This discrepancy might be explained by the 10-fold higher potency of NS4591 on the K_Ca_3.1 channel over the K_Ca_2.3 channel and that in our study NS309 preferentially used the up-regulated K_Ca_2.3 channel expression, as described above, to produce a stronger relaxation. However, NS309 in the present study produced a K_Ca_2.3/K_Ca_3.1-independent relaxation. These small dilator effects could be related to the fact that NS309 at concentrations above 10 µM blocks voltage-dependent Ca^2+^ channels [50] further corroborating substantial unspecific vasodilator effects of this compound. This is further supported by our observation that the NS309-induced relaxation in normoxic wild type mice was insensitive to the K_Ca_2.3 and K_Ca_3.1 channel blockers, apamin and charybdotoxin, which contrasts with earlier findings in rat arteries [26], [39]. Nevertheless, the increased NS309-evoked relaxation in chronic hypoxic mice were sensitive to the K_Ca_2.3 and K_Ca_3.1 channel blockers, suggesting that under these conditions and in keeping with the up-regulation of K_Ca_2.3 in the hypoxic mice, a substantial portion of vasodilator capacity is now carried by activation of the K_Ca_2.3 channel and EDH-vasodilator pathways. This interpretation is further supported by the notion that NS309-induced relaxations were not reduced by L-NNA in these hypoxic mice.

### Pulmonary hypertension in mice with genetic deficit of K_Ca_2.3 and K_Ca_3.1 channels

The K_Ca_3.1^−/−^/K_Ca_2.3^T/T(+DOX)^ mice developed increased right ventricular systolic blood pressure as well as increased right ventricular hypertrophy ratio to a similar degree as the hypoxic wild type mice. Furthermore, increased ratios of right ventricular wall thickness/heart weight was found in normoxic and hypoxic K_Ca_3.1^−/−^/K_Ca_2.3^T/T(+DOX)^ mice. This may suggest that the down-regulation of the channels caused hypertrophy of the right heart. In addition, previous studies found that the K_Ca_2.3^T/T^ mice had structural alterations, including enhancement of arteries and enlargement of other hollow organs [21], [51], thus the life-long overexpression of the channel in this genetic model of channel overexpression(-Dox)/suppression(+Dox) may influence cardiac development and morphology.

K_Ca_2.3 mRNA expression was present in Dox-treated K_Ca_3.1^−/−^/K_Ca_2.3^T/T^ mice though expression was significantly lower than in wild types, reflecting the fact that the K_Ca_2.3 channel was suppressed rather than knocked out. K_Ca_3.1 channel expression was almost abolished in K_Ca_3.1^−/−^/K_Ca_2.3^T/T(+DOX)^ mice. It is noteworthy that the genetic alterations did not affect the mRNA expression of the other Ca^+^-activated K^+^ channels (K_Ca_2.1, K_Ca_2.2, and K_Ca_1.1) compared to wild type mice and, thus, there was no evident compensation caused by increased channel expression of these related K_Ca_ channels.

Genetic deficit of K_Ca_2.3 and K_Ca_3.1 channels in normoxic and hypoxic K_Ca_3.1^−/−^/K_Ca_2.3^T/T(+DOX)^ mice increased lumen diameter in partially muscularized- and muscularized vessels compared to normoxic and chronic hypoxic wild type mice. This could be due to the structural alterations previously seen in the K_Ca_2.3^T/T^ mice [21]. Furthermore, the wall area was significantly decreased in K_Ca_3.1^−/−^/K_Ca_2.3^T/T(+DOX)^ mice compared to wild type mice in vessels of 20–39 µm. Intuitively, one would expect that these structural alterations decrease pulmonary pressure. However pulmonary vascular remodeling by itself is not necessarily sufficient for altering pulmonary pressure in chronic hypoxic mice [52]. In the present study the blood pressure was also not altered in the K_Ca_3.1^−/−^/K_Ca_2.3^T/T(+DOX)^ mice compared to wild type mice regardless of normoxic or chronic hypoxic conditions. This can probably be explained by early developmental alterations in this model.

In a previous study, K_Ca_3.1^−/−^ mice had reduced acetylcholine-induced relaxation in carotid arteries [19]. Similarly, transgenic K_Ca_3.1^−/−^/K_Ca_2.3^T/T(+DOX)^ mice exhibited defective acetylcholine-induced relaxation in carotid- and systemic resistance arteries [22]. Contrary to these previous findings, K_Ca_3.1^−/−^/K_Ca_2.3^T/T(+DOX)^ mice in the present study did not show impaired acetylcholine-induced relaxation in pulmonary arteries compared to wild type mice. However, this might be explained by the fact that the acetylcholine-induced relaxation in the murine pulmonary arteries was almost entirely mediated by NO and insensitive to blockers of K_Ca_2.3 and K_Ca_3.1 channels, while in other vascular beds the K_Ca_3.1/K_Ca_2.3-EDH-system and a possible interaction of the EDH with the NO system were found to be more important [22], [53].

Surprisingly, exposure to chronic hypoxia converted the acetylcholine-induced relaxation in these mice into an apamin- and charybdotoxin-sensitive relaxation. This could be due to an up-regulation of an apamin-sensitive K^+^ channel and/or a charybdotoxin-sensitive K^+^ channel (such as K_Ca_1.1), as an adaptation to the hypoxic condition in the K_Ca_3.1^−/−^/K_Ca_2.3^T/T(+DOX)^ mice.

In conclusion, chronic hypoxia increased expression of K_Ca_2.3 and K_Ca_1.1 genes, decreased the expression of K_Ca_2.1, and improved the endothelium-dependent vasodilatation to pharmacological activation of the channels. Such a mechanism could be of help to compensate for the loss of NO-activity in pulmonary hypertension and could be pharmacologically targeted to counteract pulmonary hypertension. Genetic deficit of K_Ca_3.1 and K_Ca_2.3 did not worsen pulmonary hypertension or endothelial dysfunction, though it prevented structural vascular remodelling in chronic hypoxia-induced pulmonary hypertension.

## Supporting Information

Figure S1 Relaxation to SNP-induced (10^−10^–10^−5^ M) in PE (10^−7^ M) pre-contracted arteries, in normoxic wild type and K_Ca_3.1^−/−^/K_Ca_2.3^T/T(+Dox)^ mice (left) and hypoxic wild type and K_Ca_3.1^−/−^/K_Ca_2.3^T/T(+Dox)^ mice.
(TIF)
