# Supplementary figures and images for: Pulmonary Hypertension in Wild Type Mice and Animals with Genetic Deficit in KCa2.3 and KCa3.1 Channels
Source: PLoS One. 2014 May 23;9(5):e97687. doi: 10.1371/journal.pone.0097687 (PMC4032241; doi:10.1371/journal.pone.0097687)

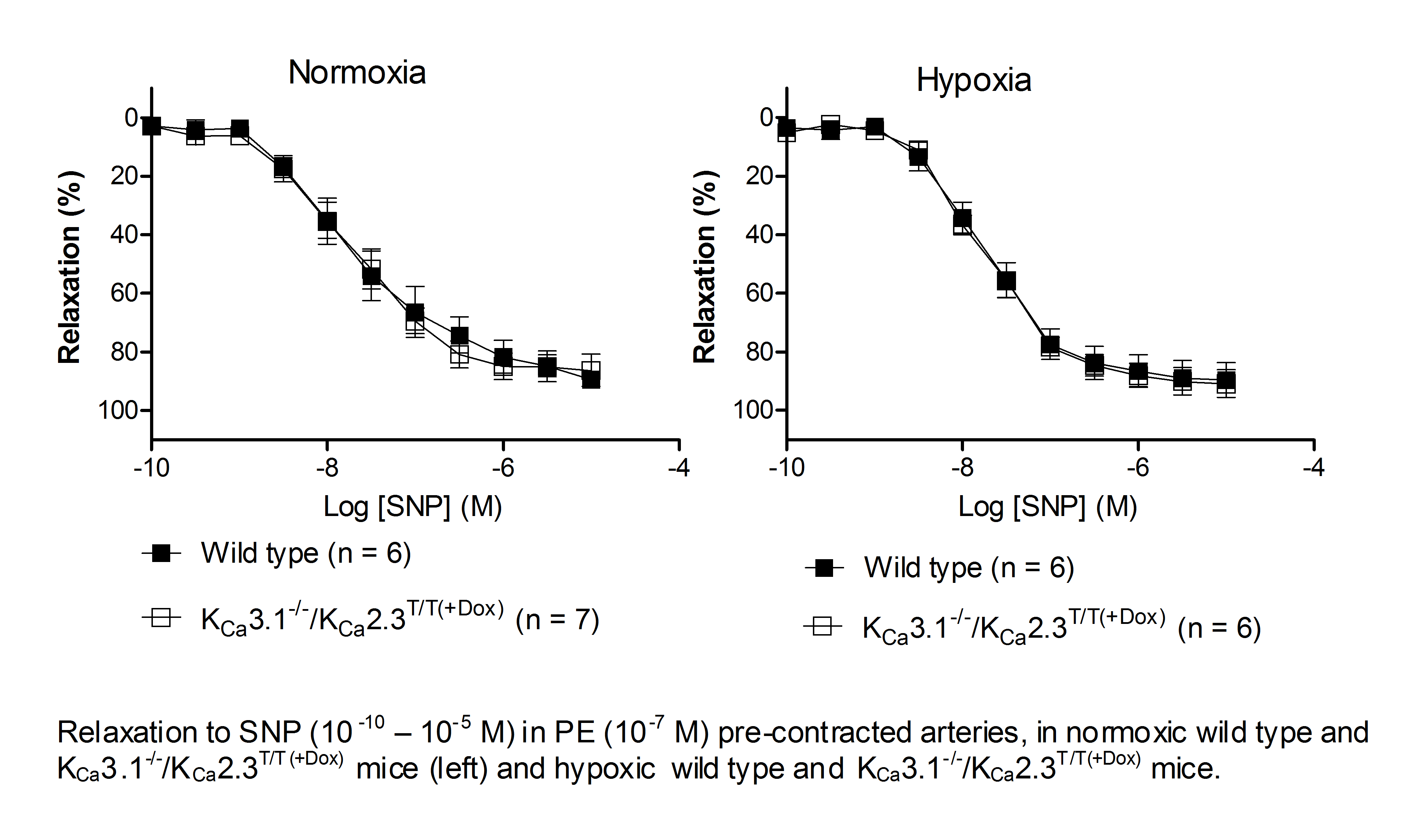

Supplement: Figure S1 — Relaxation to SNP-induced (10−10–10−5 M) in PE (10−7 M) pre-contracted arteries, in normoxic wild type and KCa3.1−/−/KCa2.3T/T(+Dox) mice (left) and hypoxic wild type and KCa3.1−/−/KCa2.3T/T(+Dox) mice. (TIF) [file pone.0097687.s001.tif]
